# Electricity Generation by *Shewanella decolorationis* S12 without Cytochrome *c*

**DOI:** 10.3389/fmicb.2017.01115

**Published:** 2017-06-20

**Authors:** Yonggang Yang, Guannan Kong, Xingjuan Chen, Yingli Lian, Wenzong Liu, Meiying Xu

**Affiliations:** ^1^Guangdong Provincial Key Laboratory of Microbial Culture Collection and Application, Guangdong Institute of MicrobiologyGuangzhou, China; ^2^State Key Laboratory of Applied Microbiology Southern ChinaGuangzhou, China; ^3^Key Laboratory of Environmental Biotechnology, Chinese Academy of SciencesBeijing, China; ^4^Guangdong Open Laboratory of Applied MicrobiologyGuangzhou, China

**Keywords:** *Shewanella*, microbial fuel cell, extracellular electron transfer, *c*-type cytochrome, *ccmA*

## Abstract

Bacterial extracellular electron transfer (EET) plays a key role in various natural and engineering processes. Outer membrane *c*-type cytochromes (OMCs) are considered to be essential in bacterial EET. However, most bacteria do not have OMCs but have redox proteins other than OMCs in their extracellular polymeric substances of biofilms. We hypothesized that these extracellular non-cytochrome *c* proteins (ENCP) could contribute to EET, especially with the facilitation of electron mediators. This study compared the electrode respiring capacity of wild type *Shewanella decolorationis* S12 and an OMC-deficient mutant. Although the OMC-deficient mutant was incapable in direct electricity generation in normal cultivation, it regained electricity generation capacity (26% of the wide type) with the aid of extracellular electron mediator (riboflavin). Further bioelectrochemistry and X-ray photoelectron spectroscopy analysis suggested that the ENCP, such as proteins with Fe–S cluster, may participate in the falvin-mediated EET. The results highlighted an important and direct role of the ENCP, generated by either electricigens or other microbes, in natural microbial EET process with the facilitation of electron mediators.

## Introduction

Bacterial extracellular electron transfer (EET) plays a crucial role in various natural biogeochemical cycles and engineering processes. It can deliver electrons from intracellular substrate to extracellular solid acceptors such as mineral oxides, humics. Moreover, bacterial EET to electrodes is a key process in the electricity generation and biodegradation in bioelectrochemical systems (BESs) which have promising application in wastewater treatment, bioremediation, biosensor and many other fields with simultaneous energy recovery (Lovley, 2006, 2012; Logan and Rabaey, 2012).

More and more bacteria (representatively *Shewanella* and *Geobacter* species) capable of EET have been isolated from various environments (Lovley, 2012). The reported bacterial EET strategies can be generally divided into two types, that is, (i) direct electron transfer to extracellular electron acceptors via outer membrane *c-*type cytochromes (OMCs) or conductively proteinaceous nanowires and (ii) indirect electron transfer via naturally occurring or biogenic electron mediators. Combined direct-indirect EET strategies can also be used by some bacteria (e.g., *Shewanella*) (Logan, 2009; Yang et al., 2012). OMCs were considered to be essential for bacterial EET as the removal of OMCs could eliminate the electron transfer efficiency in both direct and indirect EET processes (Shi et al., 2009; Coursolle et al., 2010). However, a recent analysis of the prokaryotic proteomes suggested that most prokaryotes do not have OMCs.

In most cases, bacteria perform EET within biofilms attached on the solid electron acceptors. It has been reported that, numerous redox species and extracellular redox proteins (e.g., flavoproteins, ferredoxins) other than OMCs exist in biofilms of pure- or mixed-species (Cao et al., 2011; Yates et al., 2016). The extracellular non-cytochrome *c* proteins (ENCP) may be generated by secretion or lysis of biofilm cells and play important roles in redox processes, cell protection and other functions. For example, proteomic analysis of the extracellular polymeric substances (EPS) of *Shewanella* sp. HRCR-1 biofilms identified hundreds of proteins (including ENCP) released from the inside or outer membrane of biofilm cells (Cao et al., 2011). Recently, Yates et al. (2016) detected proteins containing Fe–S clusters in BES electrode biofilms. In contrast to the well-known role of OMCs in EET, whether bacterial ENCP participates in EET or not is yet unknown. It has been reported that many bacteria lacking of OMC and EET capacity could obtain EET capacity by providing electron mediators such as neutral red or flavins (Chung et al., 1978; Park and Zeikus, 2000). Therefore, we hypothesized that ENCP could contribute to bacterial EET processes with the facilitation of electron mediators.

To verify the hypothesis, this study investigated the EET capacities (including electrode, iron and azo dye reduction) of *Shewanella decolorationis* S12 and its mutant lacking of OMCs in the presence or absence of artificial electron mediator. The results suggested a significant role of *Shewanella* ENCP in the mediated EET process. This is the first evidence of that bacterial could use not only OMCs but also ENCP in EET process. Due to the ubiquity of ENCP and electron mediators in natural and engineering environments, bacterial ENCP may play an important but unrecognized role in many EET-dominated processes and bioreactors such as dissimilatory metal reduction and BESs.

## Materials and Methods

### Bacterial Strains

*Shewanella decolorationis* S12 was isolated from the activated sludge of a textile-wastewater treatment plant (Xu et al., 2005). It has been reported that the *ccmA* gene is essential for the maturation of the *c*-type cytochromes of *Shewanella* species (Ahuja and Thony-Meyer, 2003; Chen et al., 2010). To obtain a mutant strain without OMCs, the *ccmA* gene of the *S. decolorationis* S12 was deleted as previously described (Chen et al., 2010). The wild-type (WT) and mutant-type (MT) *S. decolorationis* S12 was aerobically grown in LB medium at 30°C until the late log-phase of the culture was achieved. The cultures were then centrifuged at 6000 × *g* for 2 min, and the collected cells were washed twice in sterilized phosphate buffer saline (PBS, pH7.2) for BES inoculation.

### MFC Assembly and Operation

Dual-chamber glass MFCs were assembled as previously described (Yang et al., 2015). Briefly, plain graphite plates (2 cm × 3 cm × 0.2 cm) were used as anodes and cathodes. An Ag/AgCl electrode (+0.197 V vs standard hydrogen electrode, SHE) was used as a reference electrode to each anode. The anode and cathode chambers were separated with a piece of Nafion 115 membrane (7.1 cm^2^). After being assembled and sterilized (115°C for 20 min), the anode chamber (120 mL) was filled with 100 mL of lactate medium (12.8 g/L of Na_2_HPO_4_, 3 g/L of KH_2_PO_4_, 0.5 g/L of NaCl, 1.0 g/L of NH_4_Cl, and lactate 10 mM, pH 6.8). To stimulate biofilm growth, 0.05% (w/v) yeast extract was added to the medium. Each cathode chamber was inoculated with 100 ml sterilized phosphate buffered saline solution (PBS, pH 7.2) containing 50 mM potassium ferricyanide. MFCs were inoculated with MT-S12 or WT-S12 with the same initial cell density OD_600_ (optical density at the wavelength of 600 nm) = 0.04. For the first 24 h, both MFCs were operated aerobically non-electricity generating (open circuit) condition by bubbling air (0.15 L/min, filtered with a 0.2 μm membrane) in the anode culture to allow biofilm and planktonic cell growth. After that, to allow electrode reduction (electricity generation) of the MT-S12 and WT-S12, the MFCs were switched to anaerobically electricity-generating (closed circuit) condition by ceasing the air-inflow and tighten the cap of anode chambers and connecting the anode and cathode via a titanium wire with a 1000 Ω resistor. The electricity of MFCs under closed circuit condition was recorded with a multimeter (Keithley 2700, module 7702). Each MFC was operated at 30°C in triplication. To test the stimulation role of electron mediators to both WT- and MT-S12, riboflavin was added (2 μM each time) to MFC anode chambers using sterilized syringes during electricity generation.

### Physiological Analyses

For the planktonic cell growth, OD_600_ of the anode cultures was periodically monitored using an UV/Vis spectrophotometer (Ultraspec 6300 pro, Amersham Biosciences). Dissolved oxygen profile in the liquid culture was measured with an oxygen microelectrode (Unisense, Denmark) as reported before (Yang et al., 2015). For the biofilm growth on anodes, biofilms were sampled and the biofilm biomass was evaluated using a protein-quantification assay as previously described (Yang et al., 2015). For the flavin concentration in planktonic culture, 3 mL of the culture liquid was centrifuged at 8000 × *g* for 2 min, and the supernatant was analyzed by a fluorescence spectrometer (LS 45, PerkinElmer) with an excitation wavelength of 440 nm and an emission wavelength of 525 nm. For the flavins in biofilms, the biofilms was rinsed in PBS and scraped with a sterilized blade, followed by blending and centrifugation of 8000 × *g* for 2 min. The supernatant was analyzed with the fluorescence spectrometer. To evaluate the contribution of the biofilm ENCP in electricity generation, the biofilm were rinsed in sterilized PBS buffer (pH 7.2) containing protease K of 10 μg/ml for 5 min (Clark et al., 2007). The shorter treatment time could partially lysed the ENCP and maintain biofilm structure as verified under confocal laser scanning microscopy (CLSM).

### Microscope and Spectroscopy Analysis

The biofilm viability and structure were observed under a CLSM after being stained with a Live/Dead BacLight staining kit (Life Technologies, L7012) (Yang et al., 2015). The analyses of surface elements and associated chemical bonds of the biofilm and cell surface were analyzed by scanning electron microscopy-energy dispersive dpectrometer (SEM-EDS, HORIBA 7962H) and X-ray photoelectron spectroscopy (XPS, Thermo K-ALPHA) with a monochromatic Al Kαsource, and the XPS data was fitted with the ‘XPS peak’ software.

### Electrochemical Analyses

Before electrochemical analysis of the MFCs, the anodic culture were purged with 0.2-μm filtered purified N_2_ to avoid the possible effects of soluble oxygen on flavin redox (Kumar and Chen, 2007). Cyclic voltammetry (CV) analysis of the MFC anodes was conducted as previously reported (Yang et al., 2014). Electrochemical impedance spectroscopy (EIS) of the MFC anodes was analyzed using an electrochemical workstation (Corrtest, China). Before analysis, the cell voltage of each MFC was controlled at their open-circuit voltages for 30 min as suggested by previous reports (Jung et al., 2012). A frequency range from 10 mHz to 100 kHz with an AC signal of ±10 mV amplitude was used. The EIS data was analyzed using Zview software and the scattered initial points were removed before data analysis as suggested by Manohar and Mansfeld (2009). The sinusoidal response was monitored which verified a stable and linear condition during the EIS measurements. According to the EIS data, an equivalent circuit (R(R_ct_CPE)) consisted of an ohmic resistance(R), followed by an electrochemical charge transfer resistance (R_ct_) in parallel with a constant phase element (CPE), was used, as suggested by reported EIS analyses for *Shewanella* anodes (Manohar and Mansfeld, 2009; Ramasamy et al., 2009; Jung et al., 2012).

## Results and Discussion

### Planktonic and Biofilm Growth of WT- and MT-Strain

**FIGURE 1:**
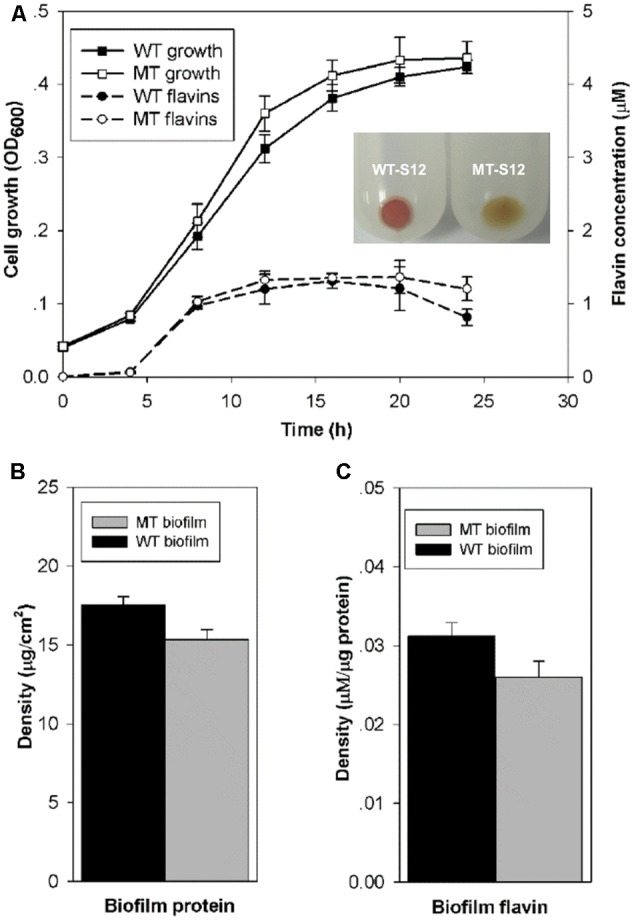
**(A)** Planktonic cell growth and flavin generation of WT- and MT-S12. Insert indicates the cell precipitate of WT- and MT-S12, wherein red color of the WT-S12 indicates cytochrome *c* on cell surface; **(B)** Biofilm biomass and **(C)** Biofilm flavins (*p* < 0.05). (*n* = 3, error bars indicate standard deviation)

Cytochrome *c* is essential for the anaerobic growth of *Shewanella* species. Physiological and molecular tests in this and our previous study demonstrated that the *ccmA*-mutant of *S. decolorationis* S12 was deficient in cytochrome *c* generation and anaerobic growth (Supplementary Figure S1) (Chen et al., 2010). To allow planktonic and biofilm cell growth of MT-S12 and WT-S12 strains, MFCs were operated aerobically for the first 24 h after inoculation which showed similar growth of the two strains (**Figure 1A**). Moreover, the flavins (including riboflavin and riboflavin-5-phosphate) generating capacity of planktonic MT-S12 and WT-S12 were comparable, indicating that cytochrome *c* had no significant effects on the aerobic growth and flavin secretion of *S. decolorationis* S12.

Regarding the biofilm growth, CLSM showed similar biofilm thickness (17 ± 7 μM) on the electrode surface of the WT-S12 and MT-S12 MFCs (Supplementary Figure S2). Despite that, the biofilm biomass of MT-S12 was less in comparison with that of the WT-S12 (15 vs 17.5 μg/cm^2^) which is consistent with the biofilm cell density observed by CLSM (**Figure 1B**). This is reasonable as the dissolved oxygen concentration decreased dramatically from the biofilm-liquid interface to the biofilm-electrode interface (from 0.08 to 0.01 mM). It is likely that the anaerobic microenvironment within the biofilm prevented the growth of MT-S12 biofilm cells as cytochrome *c* are needed in *Shewanella* anaerobic growth. In line with the biomass content, flavin concentrations in MT-S12 biofilm are lower than that in the WT-S12 biofilms. By normalizing to biofilm biomass, it can be seen that the flavin-secretion capacity of MT-S12 biofilm cells was 12.9% lower than that of WT-S12 (0.027 vs. 0.031 μM per mg protein, **Figure 1C**). Several previous reports have indicated that flavin secretion capacity of *Shewanella* species would decreased in unfavorable growth conditions as lower flavin generation was observed in anaerobic or single electron acceptor condition than in aerobic or multiple electron acceptor condition, respectively (von Canstein et al., 2008; Brutinel and Gralnick, 2012; Wu et al., 2012). It seemed likely that MT-S12 suffered more stress in the micro-aerobic biofilm environment relative to WT-S12. Moreover, the decreased flavin secretion of WT-S12 would save more energy for some other essential metabolisms to survive under unfavorable conditions (Marsili et al., 2008).

### Electricity Generation Capacity of MT and WT Strain

**FIGURE 2:**
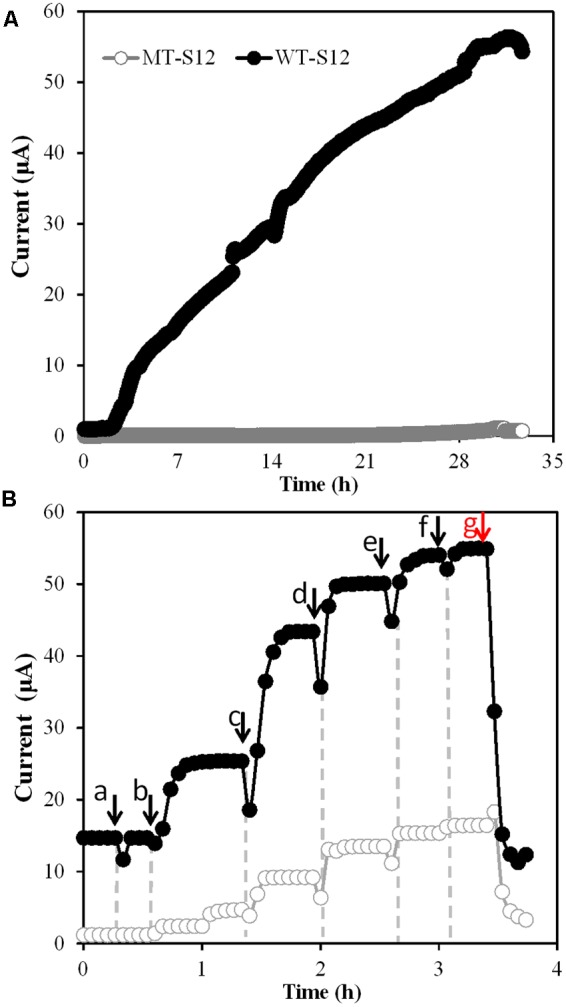
**(A)** Electricity generation by MT- and WT-S12 under normal condition. **(B)** Electricity generation by MT- and WT-S12 with increased riboflavin concentration, arrow a indicates addition of flavin-free PBS, arrow b to f indicates addition of 2 μm flavins for each, red arrow g indicates protease K treatment.

After open-circuit aerobic growth, MFCs were switched to closed-circuit anaerobic condition which allowed the WT and MT strains to use electrode as the sole electron acceptor. Electricity generation by WT-S12 started within 3 h upon switch and increased to the maximal value of 56.4 μA within 33 h (**Figure 2A**). In contrast, MT-S12 MFCs (**Figure 2A**) and abiotic controls (Supplementary Figure S3) showed no obvious electricity generation, indicating that cytochrome *c*-deficient MT-S12 lost the electrode respiration capacity. This is predictable as cytochrome *c* has been demonstrated to be essential in EET capacity of *Shewanella* and other reported microbes capable of electrode respiration (Coursolle et al., 2010; Lovley, 2012).

Electron mediators such as neutral red, flavins or AQDS are usually used to stimulate EET processes of bacteria capable or originally incapable of EET (e.g., *Escherichia coli, Actinobacillus succinogenes*) (Chung et al., 1978; Park and Zeikus, 2000). *Shewanella* species can secrete flavins as electron mediators or cofactors to deliver electrons from OMCs to electrode and stimulate the EET rate by up to 50-fold (Coursolle et al., 2010; Okamoto et al., 2014b). On the other hand, it was reported that over 300 proteins (including OMCs and various ENCP) existed in the EPS of *Shewanella* sp. HRCR-1 biofilm (Cao et al., 2011). The incapability in electricity generation of MT-S12 (**Figure 2A**) suggested that the ENCP has no significant interaction with the electrode, directly or indirectly via flavins, under normal cultivation condition.

However, when we increased the flavin concentration from 0 to 10 μM in both WT- and MT-S12 MFCs, the electricity generated by WT-S12 increased from 15.2 to 55.4 μA as expected (Okamoto et al., 2014a) and intriguingly, the electricity generated by MT-S12 increased to 14.4 μA (26% of the WT-S12) (**Figure 2B**). In addition to electricity generation, riboflavin-stimulated EET were also founded in Fe(III) and amaranth (an cell membrane-impermeable azo dye; Hong et al., 2007) reduction of MT-S12 biofilms (Supplementary Figure S4). These results suggested that although OMCs were eliminated, the electron transfer from MT-S12 to electrode could also be stimulated by higher concentration of electron mediators. This is consistent with the fact that many OMC-free bacteria could perform EET with artificially added electron mediators. Moreover, the remained slight Fe(III) and amaranth reduction capacity of MT-S12 (Supplementary Figure S4) indicated that some extracellular redox species other than OMCs exist.

### Electrochemical Interaction between Electrode and MT- and WT-S12

**FIGURE 3:**
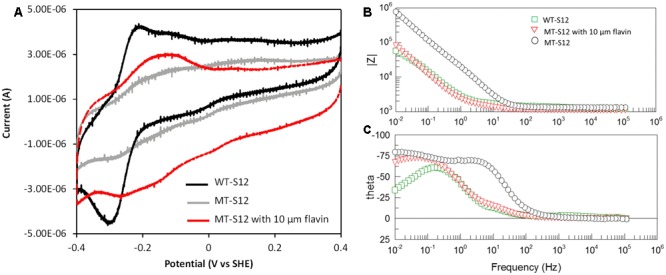
**(A)** CV of biofilms in different anodic cultures. Scan rate = 0.01 V/s, pH = 0.68. **(B)** Bode-diagram of EIS analysis. **(C)** Phase angle (theta)-frequency plots of EIS analysis, with an AC voltage amplitude of 10 mV and a frequency range from 100 mHz to 100 KHz.

To further confirm and understand the interaction between electrode and MT-S12 with artificially added riboflavin, CV, and EIS analyses were conducted. As a control, the abiotic electrode CV showed no obvious redox peak and riboflavin showed a reduction peak at –0.23 V which is consistent with previous reports (Marsili et al., 2008) (Supplementary Figure S3). WT-biofilms have a couple of reductive and oxidative peaks at –0.29 and –0.21 V (centered at –0.25 V) (**Figure 3A**), which could be attributed to flavins as cofactors of OMCs (Marsili et al., 2008; Okamoto et al., 2014b). A much wide redox area without obvious peak can be seen for MT-biofilms. After adding 10 μM of ribolavin, a wide redox couple (centered at –0.195 V) were observed. The enhanced wide-range redox peak of the MT-biofilms with added riboflavin indicated that various redox species may react with the electrode via riboflavin, which is different with the sharp CV peaks caused by the OMC-flavin of the WT-S12. It has been reported that flavins in *Shewanella* EET mainly functioned as cofactors of OMCs (one-electron reaction) which could largely enhance the EET rate than free flavins (two-electron reaction) (Kumar et al., 2008; Okamoto et al., 2014b). For the MT-S12 biofilms, flavins could only functioned in a free state due to the deletion in OMCs. Therefore, the WT-S12 biofilms showed higher anodic peak current than that of the MT-S12 biofilms with or without artificially added riboflavin.

Electrochemical impedance spectroscopy measurements of the anodes were shown in Bode-diagram (**Figure 3B**). The phase angle (theta)-frequency plots (**Figure 3C**) suggested an one-time constant model of the anodic reactions which is consistent with the used equivalent circuit (R(RctCPE). The phase angels of different anodes occurred with low frequencies (<100 Hz) indicating that the anodic reaction was dominated by flavin-mediated electron transfer processes (Ramasamy et al., 2009; Jung et al., 2012). This is consistent with the physiological analysis with several *Shewanella* mutants that flavins accounts for ∼75% in *Shewanella* EET to solid electron acceptors (Kotloski and Gralnick, 2013). The impedance modulus (Z)-frequency plots (**Figure 3B**) showed an impedance order of the three anodes: MT-S12 > MT-S12 with flavins > WT-S12 (detailed fitting data shown in Supplementary Table S1), which is consistent with their CV profiles and electricity generation capacities. Electrochemical analyses further confirmed that riboflavin could increase the charge transfer between *S. decolorationis* S12 and electrode in the presence or absence of OMCs.

### Possible Role of ENCP in MT-S12 Electricity Generation

Considering that the added flavins can only deliver extracellular electrons (Richter et al., 2010) and MT-S12 have no OMCs, it can be presumed that flavins could receive electrons from ENCP or other redox species in MT-S12 biofilm EPS and further transfer them to terminal electron acceptors.

To verify whether biofilm ENCP played a role in the flavin-enhanced electricity generation, proteinase K, a non-redox enzyme capable of destroying extracellular proteins but not intracellular proteins, was used to treat the biofilms (Clark et al., 2007). After treatment, electricity generation by MT-S12 biofilm dramatically decreased by 84% (to 2.3 μA, **Figure 2B**) which suggested that ENCP played an important role in the flavin-enhanced electrode respiration by MT-S12.

**FIGURE 4:**
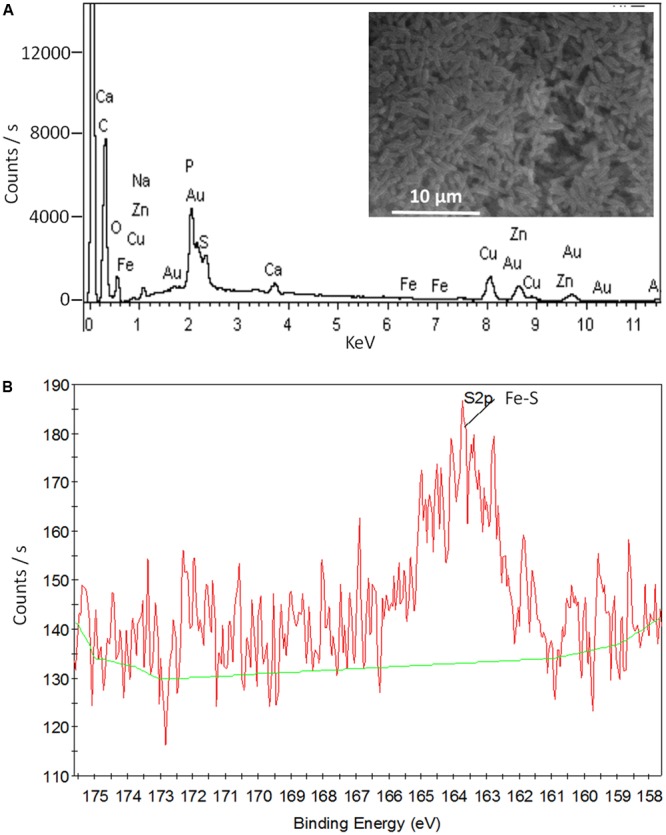
SEM- EDS **(A)** and XPS **(B)** data of the MT-S12 cell surface. Background data was shown in Supplementary Figure S5.

*Shewanella* EPS contains a plenty of ENCP that might donate electrons to flavins. Among those ENCP, Fe-S cluster containing-proteins exist widely in the EPS of different bacteria species (Cao et al., 2011; Yates et al., 2016). By using EDS, we identified element Fe in the EPS of both WT-S12 and MT-S12 (0.11% and 0.07% w/w, respectively) (**Figure 4A**). Sulfur as a common element in bio-molecular was also detected. Furthermore, the XPS peak at 163.5 eV could be attributed to Fe–SH bond (Volmer-Uebing and Stratmann, 1992) (**Figure 4B**), suggesting that Fe–S cluster exists in the EPS. Fe–S cluster can donate electrons to flavins (Watanabe et al., 2016), therefore, it is possible that Fe–S cluster-containing proteins (or other SNCP) donate electrons to the riboflavin and further to electrode. Consistently, Yates et al. (2016) recently reported that extracellular Fe–S cluster containing-proteins from *Marinobacter* spp. might play an important role in EET of an electrode attached mixed-species biofilm. To further elucidate the role of Fe–S cluster-containing proteins or other ENCP in EET, high throughput sequencing and molecular interaction analysis are needed.

Outer membrane *c*-type cytochromes-free microbes are generally considered to participate indirectly in electricity generation by providing fermentation or pre-degradation products to electricity-generating microbes rather than donating electrons to electrode (Logan, 2009; Hodgson et al., 2016). In contrast, our results indicated that the ENCP from OMC-free microbes (e.g., fermentation microbes) could also contribute to EET via electron mediators. Moreover, the contribution of OMC-free microbe in electricity generation could partially explain a repeatedly reported phenomenon that scarce or even no typical electricity generating microbes could be founded in the anode microbial communities of many well-performed BESs (Xia et al., 2015; Xiao et al., 2015).

## Conclusion

The electricity generation capacities and bioelectrochemical properties of wild *S. decoloationis* S12 and an OMC-mutant were comparatively analyzed in this study. In comparison with the wild strain, the mutant showed similar planktonic growth but slightly decreased biofilm growth. Similarly, to the wild strain, the EET rate of the OMC-mutant could be stimulated with artificially added flavins. ENCP in the biofilm EPS, such as Fe–S cluster containing-proteins, may play an important role in the EET process of the OMC-mutant. The results indicated an unignorable role of ENCP in bacterial EET process with the facilitation of electron mediators.

## Author Contributions

YY, MX, WL designed the study; YY, GK operated the experiments, XC made the mutant, YL discussed the results, YY, WL wrote the paper, all authors agree to be accountable for the content of the work.

## Conflict of Interest Statement

The authors declare that the research was conducted in the absence of any commercial or financial relationships that could be construed as a potential conflict of interest.
