# Supplementary material for: Electricity Generation by Shewanella decolorationis S12 without Cytochrome c
Source: Front Microbiol. 2017 Jun 20;8:1115. doi: 10.3389/fmicb.2017.01115 (PMC5476703; doi:10.3389/fmicb.2017.01115)
Supplement: Supplementary file 1 [file Data_Sheet_1.DOCX]

**Supplementary materials for**

**Electricity generation by** ***Shewanella decolorationis* S12 without cytochrome *c***

Yonggang Yang^1^, Guannan Kong^1^, Xingjuan Chen^1^, Yingli Lian^1^, Meiying Xu^1,2*^, Wenzong Liu^3*^

^1^ Guangdong Provincial Key Laboratory of Microbial Culture Collection and Application, Guangdong Institute of Microbiology, Guangzhou, China, ^2^ Guangdong Open Laboratory of Applied Microbiology, Guangzhou, China, ^3^ State Key Laboratory of Applied Microbiology Southern China, Guangzhou, China, ^4^ Key Laboratory of Environmental Biotechnology, Chinese Academy of Sciences, Beijing, China

**Correspondence:** Guangdong Institute of Microbiology, [xumy@gdim.cn](mailto:xumy@gdim.cn) (M Xu); Chinese Academy of Sciences, [47723557@qq.com](mailto:47723557@qq.com) (W Liu).

**Table 1** Fit-parameters for the EIS spectra of the anodes using the equivalent circuit of R(RctCPE).

|  | MT-S12 | MT-S12 + 10 μm Flavin | WT |
| --- | --- | --- | --- |
| R1(ohm) | 1315.0 | 1116.0 | 1127.0 |
| C1(F) | 1.40E-04 | 1.30E-04 | 1.02E-04 |
| R2(ohm) | 9.50E+15 | 4.07E+05 | 6.24E+04 |


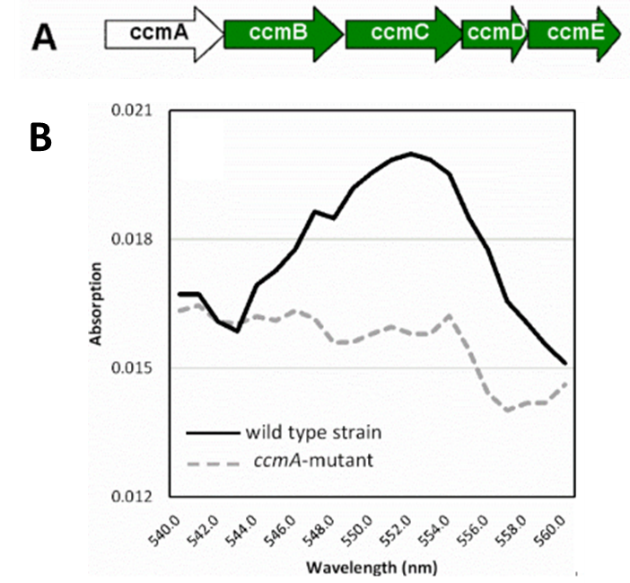


**Fig. S1**. A, the *ccm* operon in *Shewanella* and a ccm*A*-mutant was use in this study; B, the representative absorption peak of cytochrome *c* at 550 nm was observed in wild strain but not in the mutant.

**
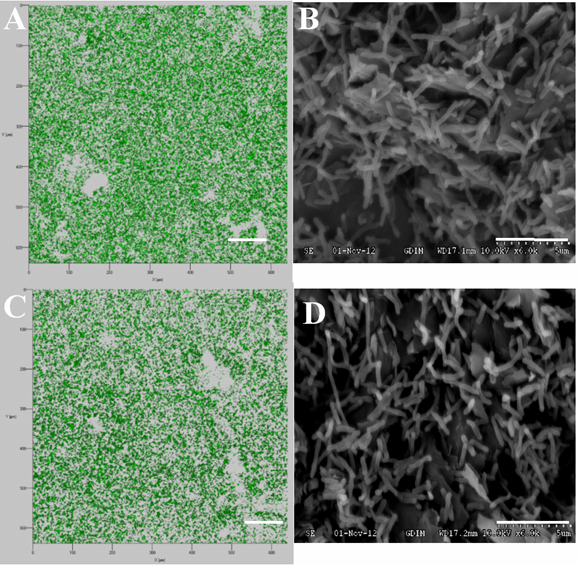
**

**Fig.S2** (A) Anode biofilm of the WT-S12 observed under CLSM. (B) Anode biofilm of the WT-S12 observed under SEM. (C) Anode biofilm of the MT-S12 observed under CLSM. (D) Anode biofilm of the MT-S12 observed under SEM.


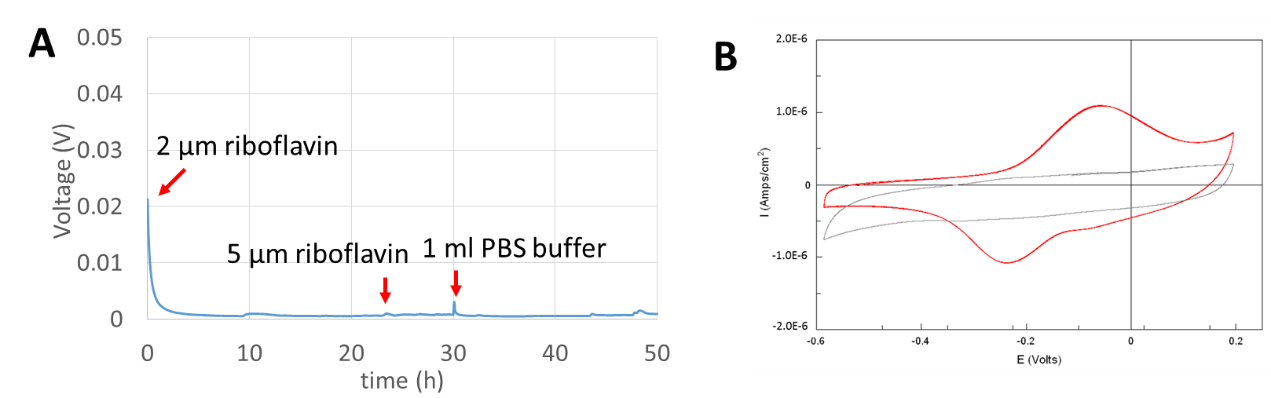


**Fig. S3** (A) Current generation of bacteria-free MFC with riboflavin addition. (B) Cyclic Voltammetry analyze of riboflavin-added (red line) and abiotic (gray line) MFC anode.





**Fig. S4** (A) Fe(III) citrate reduction by WT- and MT-S12 in the presence and absence of 5 μm riboflavin. (B) Azo dye amaranth reduction by WT- and MT-S12 in the presence and absence of 5 μm riboflavin.

**
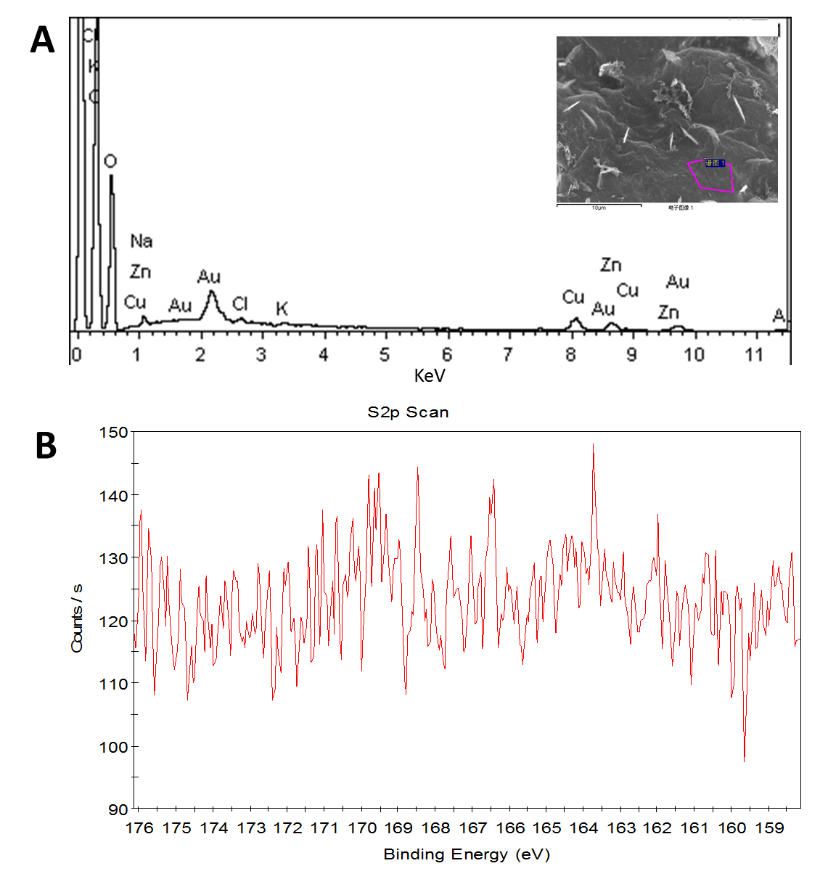
**

**Fig. S5** SEM-EDS (A) and XPS (B) data of the background materials. No Fe and S and Fe-S bond was detected in background materials suggested the elements and Fe-S bond detected in Fig. 4 could be attributed to bacterial cells.
